# Consoling the Bereaved: Exploring How Sympathy Cards Influence What People Say

**DOI:** 10.1177/00302228211065958

**Published:** 2022-01-26

**Authors:** Kimberly A. Calderwood, Amy M. Alberton

**Affiliations:** 1 Department of Social Work, 6515 Trent University Durham, Oshawa, ON, Canada; 2 School of Social Work, Wichita State University, Wichita, KS, USA

**Keywords:** sympathy cards, bereavement, condolences, consolation, discourse analysis

## Abstract

In a sequential mixed methods study, interview data showed that bereaved parents consistently reported “stupid” or “insensitive” things people said that were not helpful when their child died. Subsequently, a discourse analysis of 170 sympathy cards was conducted to assess societal messaging that may influence people’s insensitive words. The findings reflected two distinct time periods in the bereavement process: a time of sadness followed by a time of peace. Possible links to insensitive things people said included: suggestions that the sad time should only last a few days, suggestions of “healing,” religious statements such as the deceased being with God or advice to pray to God, and notions of celebration in some contexts. Very few excerpts were similar to the advice-giving quotes that interviewees considered to be insensitive. Many cards reflected the love and memories inherent in creating a continuing bond with the deceased and achieving peace.

Although there is an abundance of research and theorizing about the bereavement process, diagnostic criteria for grief (e.g., complicated, persistent complex, disturbed, and pathological), and possible assessment and treatment options for grief, relatively little has been written about the role of lay people in supporting the bereaved (Dyregrov, 2006). This is especially so with respect to how friends, family, and other informal support providers attempt to console the bereaved (Klass, 2014). Increasingly, the literature is showing that bereaved people, particularly bereaved parents, find insensitive things people say to be harmful in their bereavement process (e.g., Calderwood & Alberton, 2021; Davidowitz & Myrick, 1984; Denhup, 2019; Hogan et al., 1994). This includes but is not limited to advice-giving, clichés (Lehman et al., 1986; McCullough, 2019; Toller, 2011), expectations of a quick recovery (Dyregrov, 2004; Lehman et al., 1986), over-protective comments, religious views, and thoughtless remarks (Dyregrov, 2004). In the context of traumatic bereavement, Dyregrov (2004) goes so far as to refer to this phenomenon as “social ineptitude” (p. 30). This is important because insensitive words have been reported to have long-lasting negative effects on the bereaved, particularly bereaved parents (Calderwood & Alberton, 2021; Dyregrov, 2004; Toller, 2011).

One method used in Western societies to attempt to console bereaved people is to give or send sympathy cards. The little research that has been conducted on the messaging in sympathy cards has focused primarily on death-denying messaging (Caldwell et al., 1998; Lippy, 1977; McGee, 1981), the role of religion (Hallett, 2017), and the degree of person-centeredness in the message conveyed (McCullough, 2019). Through an analysis of 226 sympathy cards received by four bereaved people, Lippy (1977) found “sympathy” to be the core theme, often expressed with qualifiers such as “deepest” sympathy, suggesting grieving the loss of someone requires greater sympathy than other life events. Thirty percent of the cards Lippy analyzed referenced God in some way: a few that He was responsible for the death, over a quarter about Him being able to support the bereaved in some way, and some about an afterlife with God. Nineteen percent were about “thinking” of the bereaved person. Eighteen percent of the cards acknowledged “the inadequacy of language to communicate the true feelings of the sender” (p. 170). Twelve percent indicated that the bereaved person’s grief was shared by others. Lippy reported that none of the cards analyzed referred to “death” and several even showed avoidance of the reality of the death.

McGee (1981) analyzed 110 American sympathy cards and found similar results to Lippy’s (1977): “sympathy,” or “sympathy” with a qualifier such as “deepest,” “heartfelt,” and “sincere” (p. 29), were common; references to God were common and considered to be helpful; euphemisms were used rather than the word “death” and “many” (p. 30) indicated the inadequacy of being able to comfort the bereaved person. A few excerpts referred to “the importance of time for healing, courage for the future, and the hope that brighter days would lie ahead” (p. 30). Caldwell et al. (1998) replicated McGee’s (1981) study with the purpose of comparing their analysis of 137 cards to McGee’s analysis from 15 years prior. Caldwell et al. reported subtle changes in sympathy card messaging since McGee’s analysis: messages seemed to be “less formal, more personal, and more like handwritten sentiments” (p. 129). Caldwell et al. interpreted this shift as indicative of efforts to improve communication in a death-denying society. Caldwell et al. also found an increase in cards showing care and concern for, and offers of help to, the bereaved. Further, religious messages seemed to have become more suggestive of the bereaved person receiving strength from their faith/beliefs as opposed to the predominant emphasis in the 1980’s about the bereaved person one day joining the deceased.

Hallett (2017) analyzed the printed text and handwritten messages on 134 sympathy cards received by a family in a small American Midwest community after the death of an elderly family member. Like the aforementioned studies, Hallett reported that variations of the word “sympathy” were most prominent (42 out of 120 pre-printed cards) and only one card contained the word “death.” Although there were many references to God, 94 of the 134 cards did not explicitly reference Him. Many of these cards did contain religious language such as “prayer.” Typically, the sender added their own hand-written statements, often religious in nature. Hallett concluded that through the cards, the community was “co-construct[ing] and reaffirm[ing] Christian identities” (p. 58).

Several authors have been testing Burleson’s (2010) person-centeredness model of comforting others, including high, moderate, and low levels of person-centeredness. For example, McCullough’s (2019) analysis of 100 American Greeting and Hallmark sympathy cards found expressions of care to be most common (a high level of person-centeredness), supporting the trend toward increased messaging about care described by Caldwell et al. (1998). Overall, more messages reflected a moderate level of person-centeredness than a high level of person-centeredness. Moderate level messages included excerpts suggesting reunification with the deceased, complimenting the bereaved or the deceased, philosophical or religious statements, and memories. High level messages included care, presence, and identifying with the bereaved person’s feelings. Only one card was about advice-giving (a low level of person-centeredness). Like Caldwell et al. (1998), Lippy (1977) and McGee's (1981) findings, McCullough also identified cards that addressed the difficulty in knowing what to say.

Although not specifically about sympathy cards but about grief messaging more generally, and still using Burleson’s (2010) model of person-centeredness as a framework, Rack et al. (2008) found that 105 bereaved young adults ranked advice-giving or minimizing the bereaved person’s feelings as generally unhelpful. Being present, showing a willingness to listen, and expressions of care and concern were considered to be helpful. Relatedly, when Wilkum and MacGeorge (2010) asked 312 college students to imagine that a grandparent had died, findings showed a perception that some types of religious expressions improved, or at least did not hinder, the sensitivity of comforting messages. Wilkum and MacGeorge concluded that support people should consider references to God (depending on the bereaved person’s religiosity) as well as person-centeredness when delivering messages to bereaved people.

## Interview Design and Analysis

This study began with two-hour individual in-depth interviews with 20 bereaved parents, and 11 service providers, in Windsor, Ontario, Canada, asking them about the bereavement process in the context of the death of a child. As reported in Calderwood and Alberton (2021), the interviewer and bereaved parents created a timeframe outline as the parent described what was happening for them from their perspective from the time of their child’s death until the present. Service providers spoke about a timeline based on their perception of bereaved parents’ experiences. Participants were asked to indicate “keywords” for each time period described and were asked to indicate what was and was not helpful for them for each time period identified. All interviews were audio-recorded and professionally transcribed verbatim. The participant demographics, details of the open, axial, and selective coding, and bereavement process findings are described in Calderwood and Alberton (2021). This paper reports on the findings about what was and was not helpful for bereaved parents in this study.

## Findings From Interviews

### Non-Judgmental Supports Were Helpful

Responses about what was helpful for participants throughout their bereavement process were consistent across all interviewees. Bereaved parents who had support from family, friends, spouses, and/or employers spoke about how important that support was for them. Responses showed that for many bereaved parents who did not have such support, they felt very isolated and lonely. All participants who had been a client of the local bereavement service spoke about the bereavement specialist as being a “lifesaver,” many going so far as to say they may not have been here today if it was not for her. Like Toller’s (2011) findings, the local peer-support group was seen to be invaluable for validating their feelings, allowing them to see that what they were feeling was not abnormal, helping them to not feel so alone, and providing them with an opportunity to talk about their child when everyone else in their world had “moved on” and expected them to have moved on as well.

Most participants talked about the role of employment in their process. For those who returned to work, they described being at work as helpful because of the routine and because of the support of co-workers. For example, one participant said,

“Both of us were very work-oriented and found the way we could [pick up the pieces and move forward] would be to just go back to work and we were not…immersed in a lot of the thoughts of him not being there all the time.”

For those who did not have work for a routine, they either described creating a routine to give them structure or wishing they had work, so they did not spend so much time thinking about their loss. For example, one participant noted, “a week later my husband went back to work. I was a stay-at-home mom, so I found it very difficult…”

Consistent with what has been found in other studies (e.g., Dyregrov, 2006; Toller, 2011), what bereaved parents described as most helpful in the early phases of the bereavement process was people assisting with everyday practicalities such as assisting with other children, helping with funeral display boards, and/or cooking for them. As one participant noted,

“I remember one of my friends brought soup over, and food and others brought flowers and food at different times. That was helpful because we did not really feel like cooking or eating, but when they brought it over it reminded us that we needed to eat.”

Overall, what was helpful included: reassurance, normalizing, being there, encouragement and support, and others being non-judgmental. As an example of just being there, one participant indicated, “The one nurse who held me and let me cry, like she didn’t say, ‘Oh, now now, you shouldn’t cry.’ She just let me cry.” Another participant said, “Anytime day or night and I could call and they [best friend and sister] would just stay with me on the phone.”

### What Was Unhelpful Was the “Stupid Things People Say”

Consistent with Denhup’s (2019) findings in interviews with six bereaved parents in the United States, every bereaved parent and service provider interviewed in this study indicated that what was not helpful was the “stupid” and/or “insensitive” things people said. As one participant indicated, “People do not know how to deal with parents. The general public doesn’t know. Even family and friends do not know how to deal with friends who have experienced this loss.” Despite our study being conducted over 1000 kilometers (680 miles) from Denhup’s study, and in a different country, participants gave the same examples as reported by Denhup, sometimes using the exact same wording. Like Denhup, we found that friends and family strived to manage or explain the death, usually through reference to God’s role. Sample quotes provided by bereaved parents included:

“Your loved one is at peace now.”

“It was God’s will.”

“God wanted another angel.”

“God never gives you anything that you can’t handle.”

“You can have another baby.”

Other examples of what was unhelpful included friends stopping their calls to the bereaved parent and people they know turning the other direction when they saw the bereaved person in public. People avoiding talking about the deceased was also indicated as being unhelpful: “People don't acknowledge my child existed.” Some people in the bereaved parents’ informal support networks avoided showing their grief altogether:

Not seeing anyone grieve, I think that was the biggest. The fact that I don’t know if it was over and done for them, or if they hadn’t grieved, or if they were holding it in, or if they were doing it in private. Just not seeing anyone grieve.

Also, like Denhup’s (2019) findings, participants indicated that people asked insensitive questions: “What else was not too helpful? Point blank questions sometimes about events and stuff that happened at that time.” Bereaved parents also noted that it was not helpful when other parents complained about their own matters, including about their own children (e.g., “I could kill that kid” when talking about their own living child). And inappropriate comparisons were made such as, “I know what you’re going through ‘cause I lost my mother’” and “I’ve gone through this too.”

An additional category emerging from our data that Denhup (2019) did not identify but that was highlighted by Toller’s (2011) interviews with 16 bereaved parents, was advice-giving not being helpful. Examples bereaved parents provided included:

“Well, if that was me, I wouldn’t-- If that was me, I would…”

“Well get involved in something.”

“Pray to God.”

“Why don’t you come out and do this with us?”

One bereaved parent described advice-giving as, “People’s way of helping [being] to divert your attention, which worked some of the time, but not other times.” Another participant indicated, “I sometimes raised my voice at my mother-in-law because she had some suggestions, and I didn’t like them.”

Most noticeable in our data, that Denhup (2019) shows as examples of managing and explaining, were the insensitive things people said that suggested a recovery conceptualization of the bereavement process:

“You’ll get better” or “Get over it” or “Move on.”

“Clean up his stuff.”

“You can pick yourself back up” or "When are you going back to work?"

“You’ll be a lot better once you get through Christmas.”

“[You will] get over it in three months” or “It’s been six months, aren’t you better yet?”

Consistent with Dyregrov’s (2004) findings, participants in the current study recognized that the speaker did not intend to be insensitive and was just trying to help: “Well-meaning people saying things that just really *weren’t* [emphasis added; laugh] helpful.” “I know that they meant well and all those kind of things.” However, as indicated by these bereaved parents and to a limited extent in the literature, well-meaning but uninformed attempts to console the bereaved can be more harmful than helpful (Macdonald, 2019). It seems that support people are aware that what they say can be harmful as the fear of saying the wrong thing has been documented in the bereavement literature for decades (e.g., Lippy, 1983).

Our findings, combined with the literature increasingly showing that insensitive words can be unhelpful for bereaved people, led us to wonder what societal messaging might be leading to such social ineptitude. As early as 1977, Lippy indicated, “the sending of sympathy cards and the messages they contain serve to foster common values among the American people” (p. 175). As such, we explored the societal messaging in sympathy cards in North America, paying particular attention to what might influence the insensitive things people say.

## Sympathy Card Design and Analysis

In searching for sympathy cards sold online, www.hallmark.com seemed to have the most cards and was known by the researchers to have a long-standing history of selling cards in North America. The content of the 170 Hallmark sympathy cards that contained English words were typed into a Microsoft Word document verbatim, with a forward slash to indicate a different page of the card. Each segment of text was coded for the message that it seemed to be conveying. This resulted in 362 excerpts that were considered to convey a message to the receiver of the card and 70 unique codes. Often, this meant several codes per card as most cards had an excerpt on the front cover and inside, and many excerpts contained multiple messages and hence multiple codes. Excerpts were grouped according to their codes with related codes grouped together. In addition, a search in Word was conducted on keywords that seemed to re-occur throughout the cards such as time, love, sympathy, care, and labels for the time periods indicated. These counts facilitated providing readers with a sense of what words occurred most frequently and assisted with grouping similar codes together. To increase the credibility of the findings, the first author engaged in several iterations of reviewing the original dataset, the coding, and the groupings, and order of groupings. In addition, the second author reviewed all of the coding, checking the accuracy of the coding, and ensuring that there were no quotes or categories that may have been misrepresented or omitted.

## Findings From Sympathy Card Analysis

Consistent with other sympathy card analyses presented in the literature, the following were commonly found throughout the cards: variations of the expression of sympathy (x77) or being “sorry” (x17); “thinking” or “thoughts” of the bereaved (x73); “pray,” “prayer,” or “praying” (x48); the word “God” (x39) and “Lord” (x17); God providing support for the bereaved (x53); the deceased now being with God (x11); and excerpts reflecting, “there are no words” (x15). Avoidance of the word “death” was also found as there was not a single instance of use of the words “death” or “died.”

What seems to only have been highlighted by Caldwell et al. (1998) in past sympathy card studies is the trend toward more cards expressing caring and concern. In the current study, the word “love” occurred more frequently than any other word (x104) reflecting, for example, love of the card giver toward the bereaved (x15), wishes for the deceased to be surrounded by love (x7), the love for the deceased lasting forever (x23), and God providing love (x18). Similarly, the word “heart” arose 74 times in excerpts such as, “Holding you in my heart.”

**Figure 1.**
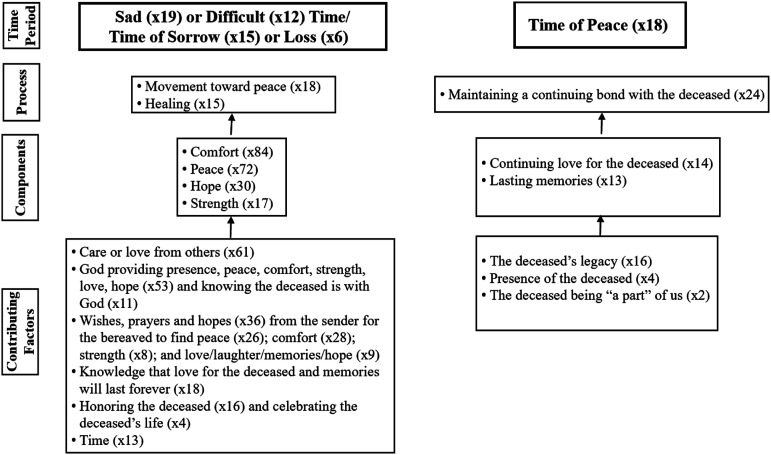
The processes, components, and contributing factors for each time period.

What we found, that was not found elsewhere in the literature, was the overwhelming emphasis throughout the cards on “time,” with the word “time” occurring 77 times plus many phrases that referred to time in other ways, such as, “days to come,” “day by day,” “today,” and “tomorrow.” Figure 1 depicts how the messaging in the sympathy cards was indicative of two distinct time periods (a sad time and a time of peace), the processes of the bereaved during each time period, the components of each process, and the contributing factors to those components.

### The Sad Time

The first of the two distinct time periods that emerged (when the card would be given) was most commonly described as a “sad time,” which arose 13 times plus six additional times when the word “sad” or “sadness” was linked with “time” elsewhere in the excerpt. This sad time was alluded to in many other ways, most frequently as a “time of sorrow” (x4) or the words “sorrow” and “time” linked somehow in the same excerpt (x11). This sad time was also referred to as a “difficult time” (x7), a “difficult journey” (x1) or “difficult days” (x1), and a “time of loss” (x6). Excerpts that exemplified this time period included the following:

“Wishing you comfort during this sad time.”

“May God’s grace bring strength, comfort, and healing in this time of sorrow.”

“…my thoughts are with you, especially during this difficult time.”

“With sympathy at your time of loss.”

#### The Process During the Sad Time

As shown in Figure 1, one process referred to during the time of sadness, sorrow, difficulty, and loss was one of striving to reach a time or place of “peace” (x18). Sample excerpts included: “Hoping each loving memory will soften the grief you feel now, bringing peace and healing day by day” and “Peace/Each day a little brighter… Each thought a little more hopeful… Each memory a little richer in comfort… Little by little. Let peace and healing come.”

As suggested in this last quote, in addition to the movement toward peace, another process during the sad time was referred to as a “healing” process (x15). Examples included:

“Hoping each loving memory will soften the grief you feel now, bringing peace and healing day by day.”

“Please know that others are with you in your grief and are here to help you on your journey toward healing.”

“And day after healing day, when you might think people have forgotten, we’ll still be caring about you.”

#### Components of Movement Toward a Time of Peace and Healing

Many excerpts referred to comfort, peace, hope, and strength as contributing factors to healing and or reaching a “time” or “place” of peace. The word “comfort” arose 84 unique times in contexts such as: comfort leading to a time of peace (x4), God providing comfort (x26), God having comfort (x1), memories bringing comfort (x14), there being comfort in knowing the deceased is now with God (x11), wishes of comfort (x29), and friends/family providing comfort (x4). One card stated, “Today… comfort: tomorrow… peace: always… memories.” Another read, “Wishing you comfort during this sad time.” Some “comfort” excerpts occurred more than once, and sometimes the word “comfort” occurred more than once in the same excerpt.

The word “peace” arose 72 times, occurring in two different ways: (1) to refer to a “time of peace” (x18), and (2) to refer to aspects of “peace” contributing to the healing process or arrival at a time (or place) of peace (x47). Contexts included: God providing peace (x15), the deceased is at peace (x4), finding or achieving peace (x7), the sender wishing the bereaved peace (x17), and “moments of peace” occurring during the sad time period (x4). Specifically related to the time of peace, one card said, “May these beautiful memories help ease your sorrow with the kind of comfort that leads the way to everlasting peace.” Referring to “peace” as contributing to healing, one card read, “Praying for you and hoping that in time peace and comfort will heal your heart.”

“Hope” was indicated 30 unique times as a contributing factor to achieving healing or peace (x7), as being provided by God (x6), and as wishes from the sender for the bereaved to have hope (x18). “Strength” was indicated 17 unique times, indicated in the context of God bringing strength (x4), others sending strength (x1), wishes for strength (x8), strength occurring over time (x2), and strength being required “to try to face tomorrow” (x1). Exemplifying hope and strength, one card stated, “In your time of sorrow may you find peace in His presence as He gives you strength for today and hope for tomorrow.” Another card read, “He gives you strength for today and hope for tomorrow.” In many cases, combinations of these words (comfort, peace, hope, and strength) were used together. For example, “But in time, the blessing of God’s unfailing grace will soften the hurt that you’re feeling and bring your heart the peace, comfort, and hope that it needs to heal” and “You’re wished strength and comfort in the days ahead.”

#### Contributing Factors to Achieving Comfort, Peace, Hope, and Strength

As shown in Figure 1, the following were referred to as contributing to the bereaved person achieving comfort, peace, hope, and strength: Care or love from others (x61); God providing presence, peace, comfort, strength, love, hope (x53) and knowing the deceased is with God (x11); wishes, prayers and hopes from family and friends (x36), specifically about the bereaved finding peace (x26), comfort (x28), strength (x8), and love/laughter/memories/hope (x9); knowledge that love for the deceased and memories will last forever (x18); honoring the deceased (x16) and celebrating the deceased’s life (x4); and time itself (x13). Each of these are described below.

As indicated above, “love” (x104) arose most frequently, and “heart” (x74) also arose frequently. The word “care” or “caring” (x28) occurred less frequently but there were 61 expressions of “kindness” or thoughtfulness toward the bereaved person, which showed care without explicit use of the word. Expressions of love were exemplified through love from the sender of the card toward the bereaved (x15), God providing love (x18), love for the deceased (x5), the deceased loving the bereaved (x2), wishes for the bereaved to be surrounded by love (x7), and expressions that love for the deceased is forever (x23). Sample excerpts showing care and love included:

“May you find comfort in the knowledge that many are holding you in their hearts today and remembering along with you.”

“Hope you know how much caring surrounds you.”

“Hope you feel the love of others around you especially now.”

“Please know that others are with you in your grief and are here to help you on your journey toward healing.”

As indicated above, the word “God” arose 39 times, “Lord” 17 times, and there were several other words representing God, such as the Almighty (x1). Related to achieving the factors that would contribute to healing or reaching a time of peace, God was portrayed as providing peace (x15), comfort (x27), love (18), presence (x20), strength (x4), and hope (x6) to the bereaved in 53 unique statements. Also, the bereaved person knowing that the deceased is now with God (x11) was suggested as being helpful. Examples relating to God included:

“Know you are being lifted up in prayer, that God would give you His gentle peace and comfort and the reassurance of His unchanging love for you”

“… He shall strengthen thine heart.”

“Hope it will give you peace to know your loved one will walk with the Father and sit at His side forever.”

Wishes, prayers, and hopes from family and friends (x36) were viewed as important contributors to the bereaved person finding peace (x26), comfort (x28), strength (x8), and love/laughter/memories/hope (x9). For example, one card said: “Hope your heart will be lifted by the love of friends and family and the memories you’re holding close.”

Knowledge that love for the deceased and memories will last forever was considered to contribute to the comfort during the sad time: “Hoping you can find some comfort in knowing the memories you shared are forever.” The word “forever” arose 14 times: 11 regarding the bereaved person’s love for and memories of the deceased lasting forever; one for the bereaved being loved by others forever, and two about the deceased being with God forever. The word “always” arose 13 times: 10 about love and memories and three about the deceased always being with God. There were eight other excerpts where “forever” was not explicitly stated but implied. Examples included:

“Nothing that is loved is ever lost.”

“May these beautiful memories help ease your sorrow with the kind of comfort that leads the way to everlasting peace.”

“Some things never end… /…like love, memory, and the legacy of a life that meant so much.”

 Excerpts specifically about memories facilitating the move from the sad time to a time of peace included:

“May these beautiful memories help ease your sorrow with the kind of comfort that leads the way to everlasting peace.”

“Hoping memories will bring you peace.”

“Hope it helps to know that beyond good-bye there is a beautiful, peaceful place where memories live forever.”

There were 16 excerpts about honoring the deceased, plus four about celebrating the deceased’s life. Some were general statements including “honor” in a list: “Keeping you close in thought as you mourn, cherish, and honor one who is so loved.” Some emphasized the contribution of the deceased. For example, “A Lasting LEGACY/Remembering someone so loved, so warm-hearted, strong and kind, recalling all the laughter shared, the love they left behind…”; and “She was here. She was loved. Her life made a difference.” There was one instance of honoring an American veteran: “HONORING A TRUE American/Your loved one’s life and service to our country will never be forgotten.….” Examples of celebration included: “Celebrating a life that touched so many…” and “Every kindness offered, each effort made in service of others, even those things merely attempted… all add up to a life worth celebrating.”

The sympathy cards highlighted the important, and sometimes gradual, role of “time” (x13) as contributing to the achievement of comfort, peace, hope and strength:

“Hoping each day that passes will let in a little more sunshine.”

“…trust in the comfort of hope… and time; ” “Hoping time will ease your sorrow.”

“Little by little, day by day, may your heartache turn into hope and your memories bring you peace.”

### The Time of Peace

The time period that followed the sad time was described as a time of “peace” (x18) and exemplified by excerpts such as, “Peace/May you find it in the days to come…” “Hoping peace gently comes to your heart”; and “Let peace and healing come.” One excerpt referred to this time as a “peaceful place”: “Beyond good-bye, there is a beautiful, peaceful place where memories live forever” (x2).

#### The Process During the Time of Peace

Unlike the movement toward peace and the healing that was described as occurring during the sad time period, there was no direct reference to a specific process during the time of peace. Also, there was no reference to any expectation of the bereaved person moving to a third time period after peace. Rather, the 24 excerpts specifically indicative of a time of peace were framed as “how it will be” after the time of healing and were dominated by words such as “always” (x14) and “forever” (x7). Examples reflecting the time of peace included: “Someday, … you will remember all you shared with less pain and more joy” and “Peace after a long journey, Love after a great loss.” Although no card spoke directly about a continuing bond, the components and contributing factors below are suggestive of maintenance of a continuing bond being the process expected during this time. As shown in Figure 1, there were far fewer excerpts about the time of peace than about the sad time.

#### Components of Maintaining a Continuing Bond

As displayed in Figure 1, the sympathy cards showed that continuing love for the deceased and lasting memories were the key components of the continuing bond process during the time of peace. Although the word “love” arose most frequently in all the cards, only 14 of these were in the context of the bereaved person’s love for the deceased lasting indefinitely. Examples included, “Forever remembered Forever loved” and “…in the loss of someone who has given you so much to remember, who will always be so dear to your heart.”

Although most excerpts related to memories were about the memories contributing to the comfort, peace, strength, and hope required to heal, 13 cards indicated that memories would be sustained even throughout the time of peace, with examples such as, “Someone who meant so much will live on in so many loving memories” and “Beyond good-bye, there is a beautiful, peaceful place where memories live forever.”

#### Contributing Factors to Achieving the Love and Memories in Continuing Bonds

As shown in Figure 1, three factors were indicated in the sympathy cards as being connected to the love and memories in the continuing bond process that would occur after healing or after the sad time. Sixteen excerpts addressed the deceased’s “legacy,” defined by Merriam-Webster (2021a) as, “of, relating to, associated with, or carried over from an earlier time....” This included: continuing connections being a result of past love for the deceased (x7); the deceased having “touched” others (x4); “leaving” a “trail” (x2), “footprints” (x2), or other things (x2); explicit use of the word legacy (x1); the deceased “giving” or “gifts” to the bereaved (x2); and three phrases such as, “A bird may fly away, but we still remember its song” and “In our hearts, there’s a smile we’ll never forget, a face we’ll always love.” The following two quotes best capture the sentiment in the above-mentioned excerpts:

“In the beauty of memories left behind, in the lives touched and changed, in the gifts of wisdom shared… those we love live on, always.”

“Some things never end…/…like love, memory, and the legacy of a life that meant so much.”

Four excerpts were about the deceased being present in the bereaved person’s life, described as “close,” “near,” or “with us.” The deceased being “a part of” the bereaved was indicated twice. For example, “The people we love become a part of who we are./And when they leave this world, a part of them stays with us…always.”

### The Uniqueness of The Bereavement Experience

Eleven excerpts were indicative of the uniqueness of the bereavement experience. Sample excerpts included:

“The time it takes to heal from a great loss is different for each of us.”

“There is no right way to grieve. So, follow your heart, feel how you feel, and know that so many people are sending you strength and wishing you peace.”

“Sending you warm thoughts and wishes right now for whatever will help you through this difficult journey.”

### Card Messages’ Connections With the Insensitive Things People Say

What was striking and aligned with the insensitive things people said, especially about quickly “getting over it,” was that the sad time was considered to be “short-lived” (x1), with better times to come “in the days ahead” or “days to come” (x11), “in the morning” or “tomorrow” (x3). Examples included:

“Wishing you strength and comfort in the days ahead.”

“Praying the pain of sorrow is short-lived.”

“Weeping may last through the night, but joy comes in the morning.”

“…may you find peace in His presence as He gives you strength for today and hope for tomorrow.”

Of all 170 cards, there were only six instances of recognition that the bereavement process was a long process. Examples included:

“Hope you know that prayers and caring thoughts will be with you for a long time to come.”

“There’s no rush to figure out what life is going to look like from now on… it will figure itself out in time.”

“Someday, not now, and perhaps not for a long while – you will remember all you shared with less pain and more joy.”

“Remember that others know your world will never be the same.”

Some phrases related to God were consistent with what interview participants considered to be insensitive, relating to the deceased now being with, or close to, God. Examples included:

“God carries them close to Him.”

“God gathers them like lambs in His arms.”

“Let’s all praise the Lord, This soul's going home in the sky… May there be great REJOICING in your loved one’s HOMEGOING.”

Although Calderwood and Alberton (2021), Davidowitz and Myrick (1984), Lehman et al. (1986), McCullough (2019), and Toller (2011) found advice-giving as particularly insensitive, only 12 of the 170 sympathy cards expressed advice (x7) or telling the bereaved what to do or what to expect (x5), with examples such as:

“Be strong, be wise, and trust that the sun will shine tomorrow.”

“This is a fragile time … a time to be gentle with yourself and give your heart permission to grieve, to feel, to remember.”

“In times of loneliness, think of all you shared together…”

“There will be things that trigger tears and things that bring laughter… things that you never expected… but they’ll gently remind you of all the wonderful details that made life with your loved one such a previous gift.”

Noteworthy, is that one card indicated, “While I might not know what advice to give, just know that I’m thinking of you,” suggesting that the bereaved might have the expectation that the support person would give advice.

## Discussion

Both the interview and sympathy card findings further confirmed that bereavement is a process. Specific to how we try to console someone who has lost a loved one, the sympathy cards suggested two time periods: the time of “sadness” “sorrow,” “difficulty,” and “loss,” followed by a time when the bereaved person had healed or had reached a time or place of peace. Components of healing or reaching a time of peace included peace, comfort, strength, and hope. Coping with loss while simultaneously working toward healing and/or peace is consistent with Stroebe and Schut’s (1999) notion of a dual process model (DPM) that emphasizes two stressors in the bereavement process: loss and restoration. In descriptions of the DPM, Stroebe and Schut (1999) acknowledge the complexity of coping regarding loss and restoration, which can include a complicated interplay of confrontation and avoidance. Consistent with other bereavement literature, Stroebe and Schut (1999) acknowledge the uniqueness of each individual’s bereavement process and do not provide a timeline. The sympathy card messaging, on the other hand, minimizes the complexity and length of the bereavement process by presenting two clear time-periods and some cards suggesting that the sad time only lasts “in the days ahead” or until “tomorrow” or “morning.” We suggest that this over-simplification risks misunderstandings that may lead to insensitive condolences relating to “getting over it” early in the process.

According to Merriam-Webster (2021b), to heal means “to become free from injury or disease: to return to a sound state.” As such, we suggest that the references to “healing” in the sympathy cards are suggestive of a recovery model of conceptualizing the bereavement process. Insensitive statements relating to healing as reported by Denhup’s (2019) and as found in our interview quotes included: “You'll get better,” “Get over it,” and “Move on.” To avoid such insensitivity, we recommend that a continuing bond approach be used instead of the healing recovery model. Consistent with the many excerpts in the sympathy cards about the bereaved person maintaining their love and memories for the deceased, the continuing bond theory posits that individuals in the grieving process hold the deceased in loving memory, maintaining an inner representation of the deceased long after they have passed (Klass et al., 1996). Rather than interpreting phrases such as, “Memories keep your loved one forever close,” as death-denying (as Lippy did in 1977), we consider such phrases to be indicative of a continuing bond that is a critical component of arriving at and maintaining peace. Our analysis showed that much less was indicated about the time of peace than about the sad time. We assume this is because sympathy cards are typically given during the sad time and are intended to console the bereaved early in the process.

Through their review of studies related to religious beliefs about death during the bereavement process, Benore and Park (2004) found that for many people, death-specific religious beliefs are crucial to adjusting to the loss. As indicated above, Hallett (2017) concluded that sympathy cards contributed to the community “co-construct[ing] and reaffirm[ing] Christian identities” (p. 58). For these reasons, religious statements clearly have a place in sympathy cards and in supporting the bereaved. However, given that religious statements may be interpreted as insensitive in some instances, as indicted by participants in the current study, it is important that the card giver consider the religiosity of the bereaved person as well as the context of the death. Statements such as, “God wanted another angel,” “It was God’s will,” or “Pray to God” are not helpful if the bereaved person does not believe in angels, God, or God’s ability to answer prayers. Similarly, comments about the deceased now being with God are insensitive if the bereaved does not believe in an afterlife. Context of the death should also be considered when referring to celebrating a life. Although the notion of a celebration may be an important contributing factor in moving from the sad time to healing or peace, we suggest that statements about celebration may not be suited in the case of the death of a young person who did not have the opportunity to experience or contribute much in their life, or in the instance of a particularly unexpected or tragic death.

## Conclusion

Like Wilkum and MacGeorge’s (2010) conclusion, we recommend that card givers, and people attempting to console bereaved people with words, consider the religiosity of the bereaved person and err on the side of being less religious if the bereaved person’s religiosity is unknown. Further, this study provides further evidence that person-centeredness, as described by Burleson (2010), should be considered, striving for high person-centeredness including care, presence, love, and sharing in the bereaved person’s sorrow. Low person-centeredness, such as advice-giving, should be avoided. Moreover, the type and circumstances of the death should be considered, as statements that may be suited in the context of an elderly person who lived a full life and died of natural causes may not be suited to the death of a young person or a particularly unexpected or tragic death. Even within type of death, it is important to recognize that each individual and circumstance is unique and hence each bereaved person will grieve in their own way. Since our interview findings and much of the literature on insensitive things people say in the context of bereavement are limited to the experience of bereaved parents primarily in North America, we recommend further research for other types of deaths and contexts.
